# The Changes in Levels and Barriers of Physical Activity Among Community-Dwelling Older Adults During and After the Fifth Wave of COVID-19 Outbreak in Hong Kong: Repeated Random Telephone Surveys

**DOI:** 10.2196/42223

**Published:** 2023-01-23

**Authors:** Zixin Wang, Yuan Fang, Paul Shing-Fong Chan, Fuk Yuen Yu, Fenghua Sun

**Affiliations:** 1 Jockey Club School of Public Health and Primary Care Chinese University of Hong Kong Hong Kong Hong Kong; 2 Department of Health and Physical Education The Education University of Hong Kong Hong Kong Hong Kong

**Keywords:** COVID-19, physical activity, older adults, barriers, changes, repeated random telephone survey, China, aging, elderly population, community-dwelling older adults, health promotion, telehealth

## Abstract

**Background:**

COVID-19 has had an impact on physical activity (PA) among older adults; however, it is unclear whether this effect would be long-lasting, and there is a dearth of studies assessing the changes in barriers to performing PA among older adults before and after entering the “postpandemic era.”

**Objective:**

The aim of this study was to compare the levels and barriers of PA among a random sample of community-dwelling older adults recruited during (February to April 2022) and after the fifth wave of the COVID-19 outbreak (May to July 2022) in Hong Kong. In addition, we investigated factors associated with a low PA level among participants recruited at different time points.

**Methods:**

This study involved two rounds of random telephone surveys. Participants were community-dwelling Chinese-speaking individuals aged 65 years or above and having a Hong Kong ID card. Household telephone numbers were randomly selected from the most updated telephone directories. Experienced interviewers carried out telephone interviews between 6 PM and 10 PM on weekdays and between 2 PM and 9 PM on Saturdays to avoid undersampling of working individuals. We called 3900 and 3840 households in the first and second round, respectively; for each round, 640 and 625 households had an eligible older adult and 395 and 370 completed the telephone survey, respectively.

**Results:**

As compared to participants in the first round, fewer participants indicated a low level of PA in the second round (28.6% vs 45.9%, *P*<.001). Participants in the second round had higher metabolic equivalent of tasks-minutes/week (median 1707.5 vs 840, *P*<.001) and minutes of moderate-to-vigorous PA per week (median 240 vs 105, *P*<.001) than those in the first round. After adjustment for significant background characteristics, participants who perceived a lack of physical capacity to perform PA (first round: adjusted odds ratio [AOR] 3.34, *P*=.001; second round: 2.92, *P*=.002) and believed that PA would cause pain and discomfort (first round: AOR 2.04, *P*=.02; second round: 2.82, *P*=.001) were more likely to have a low level of PA in both rounds. Lack of time (AOR 4.19, *P*=.01) and concern about COVID-19 infection during PA (AOR 1.73, *P*=.02) were associated with a low level of PA among participants in the first round, but not in the second round. A perceived lack of space and facility to perform PA at home (AOR 2.03, *P*=.02) and unable to find people to do PA with (AOR 1.80, *P*=.04) were associated with a low PA level in the second round, but not in the first round.

**Conclusions:**

The level of PA increased significantly among older adults after Hong Kong entered the “postpandemic era.” Different factors influenced older adults’ PA level during and after the fifth wave of the COVID-19 outbreak. Regular monitoring of the PA level and its associated factors should be conducted to guide health promotion and policy-making.

## Introduction

Hong Kong has a rapidly aging population. By 2030, 22% of Hong Kong residents will be ≥65 years old [1]. Recent data show that 75% of older adults in Hong Kong are suffering from one or more chronic diseases [1]. This situation has already created a huge burden on the local health system [1]. Physical activity (PA) is defined as any bodily movement produced by the skeletal muscles that results in an expenditure of energy, and is widely recognized as an effective intervention for reducing mortality and the risk of dependence-inducing diseases in older adults [2]. Systematic reviews have shown that sustainable PA improved cognitive functions, frailty symptoms, body composition, and physical functions among older adults [3,4]. Therefore, the World Health Organization (WHO) recommends that older adults without any contradiction to PA should perform at least 150 minutes of moderate-intensity aerobic PA, at least 75 minutes of vigorous-intensity aerobic PA, or an equivalent combination of moderate-to-vigorous PA (MVPA) every week [2]. Local health authorities follow the same recommendation.

Physical inactivity remains a global phenomenon and increases significantly with age. A systematic review showed that 43.4%-78.0% of older adults across countries could not meet the WHO-recommended PA level [5]. In Hong Kong, the prevalence of physical inactivity was 13.5% among people aged 65-74 years, 22.4% among those aged 75-84 years, and 42.8% among those aged 85 years or above in 2019 [6]. Another study conducted in Hong Kong oHbefore the COVID-19 outbreak reported that 20% of individuals aged ≥60 years had a low PA level [7]. Globally, the COVID-19 pandemic has negatively affected PA levels among older adults [8]. As compared to the time before COVID-19, studies consistently observed a decline in PA level among older adults after the COVID-19 outbreak [9-18]. Similar trends were observed in Hong Kong. As compared to the PA situation in 2019, two studies found a decline in the frequency of walking and moderate- and high-intensity sports among the general population, and in the overall PA level among men aged ≥60 years after the COVID-19 outbreak [10,19]. Therefore, there is an urgent need to improve PA among older adults in Hong Kong, especially considering the negative impact of COVID-19.

Several different facilitators and barriers affect the participation of PA among older adults. A systematic review suggested that lack of knowledge, skills, capacities, and support from peers or family members related to PA; perceived cons of PA (causing pain, risk of injury, and fear of falling); and environmental barriers (access to facilities and transportation, bad weather) were the main barriers of performing PA among community-dwelling older adults [20]. Perceived benefits of PA (improved physical and mental health, fun and enjoyment), perceived self-efficacy, and suggestions from health professionals were highlighted as facilitators [20]. Similar facilitators and barriers applied to Hong Kong older adults before the COVID-19 outbreak [7]. During the outbreak, COVID-19 control measures (closure of exercise facilities, social distancing) significantly increased older adults’ difficulties in accessing sports facilities and reduced support from peers [8]. An increase in caring responsibility during the pandemic due to school closure further reduced the availability for PA [21]. Moreover, concerns about the risk of COVID-19 infection reduces older adults’ motivation, and increases fear and anxiety related to PA [8,16,18]. Achieving “zero-COVID” is difficult. Instead, most countries have started to relax COVID-19 control measures and “return to normal” with high COVID-19 vaccination coverage. In Hong Kong, the government lifted its strict COVID-19 control measures (eg, closure of schools and sports facilities, prohibiting group gatherings) when the number of daily new cases dropped to about 300 (April 2022) [22]. Despite an increasing trend of daily confirmed COVID-19 cases (from <300 in May 2022 to over 3000 in July 2022), the government did not tighten up COVID-19 control measures again [22]. The changing pandemic and its control measures might influence barriers to performing PA among older adults. To our knowledge, it remains unclear whether the impact of COVID-19 on PA among older adults would be long-lasting, and there is a dearth of studies assessing the changes in barriers to performing PA among older adults before and after entering the “postpandemic era.”

To address the above-mentioned knowledge gaps, we analyzed the data of two rounds of cross-sectional random telephone surveys among community-dwelling older adults in Hong Kong, China. We compared levels and barriers to performing PA between rounds. In addition, we investigated the factors associated with a low PA level among participants of different rounds. We hypothesized that less participants would have a low PA level in the second round of the survey compared to the first round. Associated factors of low PA level were also expected to be different between the two rounds of the survey.

## Methods

### Study Design

**Figure 1:**
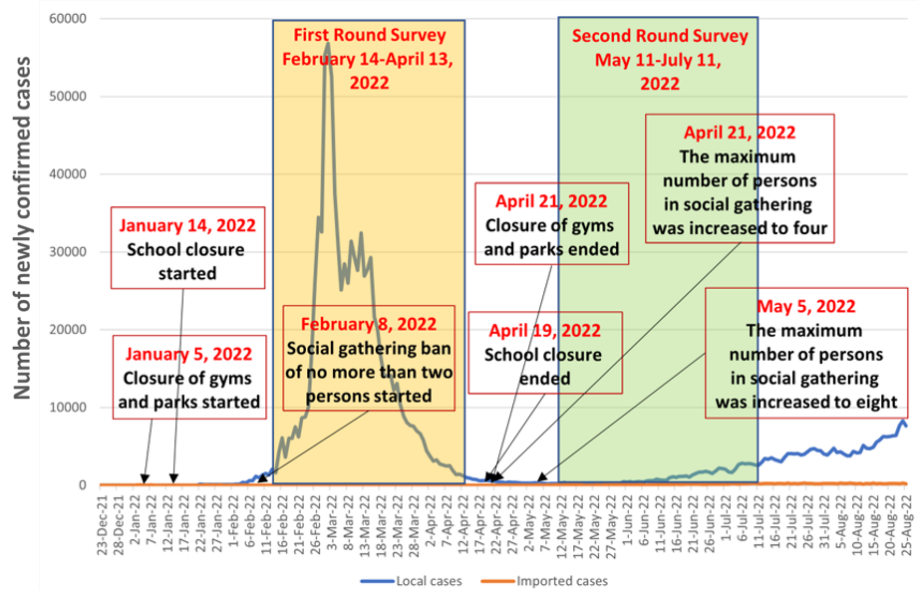
The COVID-19 situation and its control measures in Hong Kong during the study period.

This study was a secondary analysis of two rounds of random telephone surveys investigating COVID-19 vaccination uptake among community-dwelling Chinese-speaking individuals aged 65 years or above in Hong Kong, China [23]. STROBE checklist for cross-sectional study was shown in Multimedia Appendix 1. The first round was conducted during the fifth wave of the COVID-19 outbreak between February 14 and April 13, 2022. During the first round, strict COVID-19 control measures were implemented [24], including (1) closure of all playgroups, kindergartens, and primary schools (between January 14 and April 19, 2022); (2) closure of fitness centers, swimming pools, and sports premises (between January 5 and April 21, 2022); and (3) prohibition of social gatherings involving more than two persons (between February 8 and April 21, 2022). The number of daily confirmed COVID-19 cases reached its peak on March 2, 2022 (n=56,827) and dropped to 1043 on April 13, 2022. The second round of telephone surveys was conducted between May 11 and July 11, 2022. The number of daily confirmed COVID-19 cases increased slowly from 294 on May 11, 2022, to 2769 on July 11, 2022. A summary of the COVID-19 situation and its control measures in Hong Kong during the study period is presented in Figure 1.

### Participants and Data Collection

Inclusion criteria of the participants were: (1) community-dwelling Chinese-speaking individuals aged 65 years or above and (2) having a Hong Kong ID card. The exclusion criterion was not able to communicate effectively with the study interviewers. We used the same data collection methods in both rounds of surveys and reported these details previously [23]. First, we input all household telephone numbers listed in the most updated telephone directories (approximately 350,000) into an Excel file. We then randomly selected 4000 numbers by using the function “select random cells.” Experienced interviewers carried out telephone interviews between 6 PM and 10 PM on weekdays and between 2 PM and 9 PM on Saturdays to avoid undersampling of working individuals. We considered a household to be nonvalid (one without an eligible participant) if no one answered five calls made at different time slots. If there was more than one individual in the household who was 65 years or above, the interviewers invited the person whose last birthday was the closest to the survey date to join the study. This practice was adopted to avoid clustering effects (ie, older adults living in the same household sharing a similar PA level and determinants of PA). Interviewers screened prospective participants for eligibility; briefed them about the study; and made guarantees of anonymity, their right to quit at any time, and that refusal to participate would have no consequences. Verbal informed consent was obtained. The interviewers also signed a form pledging that the participants had been fully informed about the study. The telephone interview took approximately 20 minutes to complete. No incentives were given to the participants.

### Ethics Approval

Ethics approval was obtained from the Survey and Behavioral Research Ethics Committee of the Chinese University of Hong Kong (SBRE-19-187).

### Measures

#### Background Characteristics

The English and Chinese versions of the questionnaires are provided in Multimedia Appendix 2. Participants reported sociodemographic characteristics (eg, age, sex assigned at birth, relationship status, education level, employment status, income, and living arrangement), presence of chronic diseases, and history of COVID-19 and COVID-19 vaccination.

#### PA Assessment

The interviewers assessed participants’ PA in the past week using the validated Chinese version of the 7-item International Physical Activity Questionnaire-Short Form (IPAQ-SF) [25]. The IPAQ-SF was validated in Chinese older adults [26,27]. The questionnaire assessed the walking, and moderate- and vigorous-intensity activities of an individual in the past week. We computed the metabolic equivalent of tasks (MET) minutes per week and minutes of MVPA per week, and categorized the PA levels into high, moderate, and low based on the protocol of IPAQ-SF [25]. A high level of PA was defined as (1) vigorous-intensity activity on at least 3 days achieving a minimum total PA of ≥1500 MET-minutes/week or (2) ≥7 days of any combination of walking or moderate- or vigorous-intensity activities achieving a minimum total PA of ≥3000 MET-minutes/week. A moderate PA level was defined as: (1) ≥3 days of vigorous-intensity activity of at least 20 minutes per day, (2) ≥5 days of moderate-intensity activity and/or walking of at least 30 minutes per day, or (3) ≥5 days of any combination of walking and moderate-intensity or vigorous-intensity activities achieving a total PA of 600 MET-minutes/week. Participants who did not meet the criteria of a moderate or high level of PA were considered to have a low PA level.

#### Barriers to Performing PA

Nine items were constructed for this study to assess barriers to performing PA (response categories: 1=disagree, 2=neutral, and 3=agree). They were: (1) do not have time, (2) lack of interest, (3) cannot find people to do PA with, (4) lack of physical capacity to do PA, (5) PA will cause pain and discomfort, (6) lack of space and facility to do PA at home, (7) concern about COVID-19 infection during PA, (8) closure of facilities due to COVID-19 and its control measures, and (9) peers refused to do PA with you due to COVID-19. Responses to these items were dichotomized (“disagree/neutral” vs “agree”) for analysis.

### Sample Size Determination

The target sample size for each round of the survey was 400. The sample size planning for the original study was explained in a previous publication [23]. In brief, assuming the proportion of participants having a low PA level of 30%-70% in the first round, this sample size was determined to be sufficient to detect a minimum between-round difference of 8.6% (power=0.80, α=.05; calculated with PASS 11.0 software).

### Statistical Analysis

There were no missing values in either round of survey. The differences in background characteristics between participants in the first and second rounds were assessed using *χ*^2^ tests. After controlling for background variables with significant differences between rounds, the differences in the level of PA, MET-minutes/week, minutes of MVPA per week, and barriers to performing PA were compared using ordinal, logistic, or linear regression models. The level of PA, MET-minutes/week, and minutes of MVPA per week between rounds were further compared in subgroups of participants with and without a specific barrier to performing PA (ie, lack of physical capacity to perform PA or perception that PA would cause pain and discomfort). The subsequent analysis was performed among participants in the same round of the survey. Using a low level of PA as the dependent variable and background characteristics as independent variables, crude odds ratios were obtained using logistic regression models. The associations between barriers to performing PA and the dependent variable were then obtained by fitting a single logistic regression model involving one of the independent variables and all significant background characteristics. Adjusted odds ratios (AORs) and respective 95% CIs were obtained. SPSS version 26.0 (IBM Corp, Armonk, NY, USA) was used for data analysis, with *P*<.05 considered as statistically significant.

## Results

### Background Characteristics

We called 3900 and 3840 households in the first and second rounds, 640 and 625 households had an eligible older adult, 245 and 255 refused to participate in the study, and 395 and 370 completed the telephone survey, respectively. The response rate was 62% and 59% in the first and second round, respectively. The characteristics of the participants are presented in Table 1. Approximately half of the participants were aged 70 years or above (first round, 50.1%; second round, 50.8%) and female. Most of the participants were married or cohabited with a partner, did not receive tertiary education (first round, 89.9%; second round, 89.2%), without full-time/part-time employment, and with a monthly household income lower than HK $20,000 (US $2580). Approximately one-fifth of the participants had at least one chronic condition (first round, 19.2%; second round, 18.9%). There was no difference in these characteristics between participants in the first and second rounds (*P* values ranged from .68 to .98). As compared to participants in the first round, more participants in the second round reported a history of COVID-19, completed the primary COVID-19 vaccination series (92.1% vs 78.7%, *P*<.001), and had received COVID-19 vaccine booster doses (58.9% vs 31.6%, *P*<.001).

**Table 1:** Background characteristics of the participants.

Characteristics				Round 1 (n=395), n (%)		Round 2 (n=370), n (%)		*P* value	
**Sociodemographic characteristics**									
	**Age group (years)**								.98
		65-69	197 (49.9)		182 (49.2)				
		70-74	132 (33.4)		125 (33.8)				
		75 or above	66 (16.7)		63 (17.0)				
	**Sex assigned at birth**								.88
		Male	157 (39.7)		145 (39.2)				
		Female	238 (60.3)		225 (60.8)				
	**Relationship status**								.79
		Currently single	97 (24.6)		94 (25.4)				
		Married or cohabiting	298 (75.4)		276 (74.6)				
	**Education level**								.94
		Primary or below	167 (42.3)		157 (42.4)				
		Secondary	188 (47.6)		173 (46.8)				
		Tertiary or above	40 (10.1)		40 (10.8)				
	**Current employment status**								.88
		Unemployed/retired/homemaker	339 (85.8)		319 (86.2)				
		Full-time/part-time	56 (14.2)		51 (13.8)				
	**Monthly household income, HK $ (US $)**								.99
		<20,000 (2580)	292 (74.3)		273 (74.2)				
		≥20,000 (2580)	53 (13.5)		49 (13.3)				
		Refuse to disclose	48 (12.2)		46 (12.5)				
	**Receiving Comprehensive Social Security Assistance (CSSA)**								.88
		No	364 (92.2)		342 (92.4)				
		Yes	31 (7.8)		28 (7.6)				
	**Living alone**								.75
		No	328 (83.0)		304 (82.2)				
		Yes	67 (17.0)		66 (17.8)				
**Medical history**									
	**Presence of chronic conditions, yes**								
		Hypertension	188 (47.6)		173 (46.8)		.82		
		Chronic cardiovascular diseases	43 (10.9)		40 (10.8)		.97		
		Chronic lung diseases	8 (2.0)		6 (1.6)		.68		
		Chronic liver diseases	8 (2.0)		8 (2.2)		.90		
		Chronic kidney diseases	2 (0.5)		2 (0.5)		.95		
		Diabetes mellitus	76 (19.2)		70 (18.9)		.91		
		Any of above							
	**History of COVID-19**								<.001
		No	353 (89.4)		276 (74.6)				
		Yes	42 (10.6)		94 (25.4)				
	**Number of doses of COVID-19 vaccination received**								<.001
		0	32 (8.1)		16 (4.3)				
		1	52 (13.2)		13 (3.5)				
		2	186 (47.1)		123 (33.2)				
		3	125 (31.6)		205 (55.4)				
		4	0 (0.0)		13 (3.5)				

### Changes in PA

The changes in PA are presented in Table 2. As compared to participants in the first round, fewer participants had a low level of PA in the second round. Participants in the second round had higher MET-minutes/week and minutes of MVPA per week than those in the first round. Subgroup analysis showed that older adults with some specific barriers to performing PA (ie, lack of physical capacity or perceived that PA would cause pain and discomfort) had a significantly lower PA level, MET-minutes/week, and minutes of MVPA per week compared to those of participants without such barriers (see Multimedia Appendix 3).

**Table 2:** Physical activity and barriers to performing physical activity.

Variables				Round 1 (n=395)		Round 2 (n=370)		*P* value				
								Unadjusted			Adjusted	
**Physical activity**												
	**Level of physical activity, n (%)**								<.001			<.001^a^
		Low	170 (45.9)		106 (28.6)							
		Moderate	147 (39.7)		161 (43.5)							
		High	53 (14.3)		103 (27.8)							
	MET^b^-minutes/week, median (IQR)		840.0 (371.3-1834.3)		1707.5 (716.6-3395.0)		<.001			<.001^c^		
	Minutes of moderate-intensity or vigorous-intensity physical activity (MVPA) per week, median (IQR)		105 (0-315)		240 (67.5-600)		<.001			<.001^c^		
**Barriers to performing physical activity, n (%)**												
	**Do not have time**								.18			.08^d^
		Disagree/neutral	368 (93.2)		353 (95.4)							
		Agree	27 (6.8)		17 (4.6)							
	**Lack of interest**								.08			.12^d^
		Disagree/neutral	342 (86.6)		303 (81.9)							
		Agree	53 (13.4)		67 (18.1)							
	**Cannot find people to do physical activity together**								.73			.29^d^
		Disagree/neutral	287 (72.7)		273 (73.8)							
		Agree	108 (27.3)		97 (26.2)							
	**Lack of physical capacity to do physical activity**								.58			.47^d^
		Disagree/neutral	355 (89.9)		328 (88.6)							
		Agree	40 (10.1)		42 (11.4)							
	**Physical activity will cause pain and discomfort**								.98			.83^d^
		Disagree/neutral	335 (84.8)		314 (84.9)							
		Agree	60 (15.2)		56 (15.1)							
	**Lack of space and facility to do physical activity at home**								.04			.02^d^
		Disagree/neutral	274 (69.4)		281 (75.9)							
		Agree	121 (30.6)		89 (24.1)							
	**Concern about COVID-19 infection when doing physical activity**								<.001			<.001^d^
		Disagree/neutral	118 (29.9)		189 (51.1)							
		Agree	277 (70.1)		181 (48.9)							
	**Closure of facilities due to COVID-19 and its control measures**								.26			.046^d^
		Disagree/neutral	232 (58.7)		232 (62.7)							
		Agree	163 (41.3)		138 (37.3)							
	**Peers refused to do physical activity with you due to COVID-19**								.10			.01^d^
		Disagree/neutral	238 (60.3)		244 (65.9)							
		Agree	157 (39.7)		126 (34.1)							

^a^*P* values obtained from multivariate ordinal logistic regression after adjusting for history of COVID-19 and number of doses of COVID-19 vaccination received.

^b^MET: metabolic equivalent of tasks.

^c^*P* values obtained from multivariate linear regression models after adjusting for history of COVID-19 and number of doses of COVID-19 vaccination received.

^d^*P* values obtained from multivariate logistic regression models after adjusting for history of COVID-19 and number of doses of COVID-19 vaccination received.

### Changes in Barriers to Performing PA

The changes in barriers to performing PA are also presented in Table 2. As compared to participants in the first round, fewer participants in the second round reported lack of space and a facility to do PA at home or being concerned about COVID-19 infection when doing PA. Fewer participants had experienced the closure of facilities and refusal by peers to do PA with them due to COVID-19 compared to those in the first round.

### Factors Associated With Having a Low PA Level

In univariate analysis, participants who completed the primary COVID-19 vaccination series and/or booster dose were less likely to have a low PA level in both rounds (Table 3). After adjustment for these significant characteristics, participants who perceived a lack of physical capacity to do PA and believed that PA would cause pain and discomfort were more likely to have low level of PA in both rounds. Do not have time and concern about COVID-19 infection during PA were associated with a low level of PA among participants in the first round, but not in the second round. Perceived lack of space and facility to do PA at home and cannot find people to do PA together were associated with a low PA level in the second round, but not in the first round (Table 4).

**Table 3:** Associations between background characteristics and low physical activity level.

Characteristics		Round 1 (n=395)			Round 2 (n=370)	
		OR^a^ (95% CI)	*P* value	OR (95% CI)		*P* value
**Age group (years)**						
	65-69 (reference)	1.0	—^b^	1.0		—
	70-74	1.40 (0.90-2.19)	.14	0.96 (0.57-1.62)		.88
	75 or above	1.32 (0.75-2.31)	.33	1.96 (1.08-3.58)		.03
**Sex assigned at birth**						
	Male (reference)	1.0	—	1.0		—
	Female	0.86 (0.58-1.29)	.48	0.98 (0.62-1.55)		.92
**Relationship status**						
	Currently single (reference)	1.0	—	1.0		—
	Married or cohabiting	1.13 (0.71-1.79)	.61	1.55 (0.89-2.68)		.12
**Education level**						
	Primary or below (reference)	1.0	—	1.0		—
	Secondary	0.86 (0.57-1.31)	.48	1.26 (0.78-2.04)		.36
	Tertiary or above	0.80 (0.40-1.61)	.54	1.58 (0.75-3.31)		.23
**Current employment status**						
	Unemployed/retired/homemaker (reference)	1.0	—	1.0		—
	Full-time/part-time	0.74 (0.42-1.32)	.31	1.16 (0.61-2.21)		.64
**Monthly household income, HK $ (US $)**						
	<20,000 (2580) (reference)	1.0	—	1.0		—
	≥20,000 (2580)	0.86 (0.47-1.54)	.60	0.86 (0.43-1.70)		.66
	Refuse to disclose	0.67 (0.36-1.26)	.21	0.66 (0.31-1.39)		.27
**Receiving CSSA^c^**						
	No (reference)	1.0	—	1.0		—
	Yes	0.64 (0.30-1.36)	.24	0.39 (0.13-1.16)		.09
**Living alone**						
	No (reference)	1.0	—	1.0		—
	Yes	1.11 (0.66-1.88)	.69	0.92 (0.51-1.67)		.79
**Presence of any chronic conditions**						
	No (reference)	1.0	—	1.0		—
	Yes	0.87 (0.58-1.30)	.49	1.13 (0.71-1.79)		.62
**History of COVID-19**						
	No (reference)	1.0	—	1.0		—
	Yes	0.99 (0.52-1.87)	.96	1.23 (0.74-2.05)		.42
**Number of doses of COVID-19 vaccination received**						
	0-1 (reference)	1.0	—	1.0		—
	2	0.59 (0.35-0.99)	.048	0.43 (0.19-0.99)		.049
	3-4	0.50 (0.29-0.88)	.02	0.48 (0.22-1.05)		.07

^a^OR: odds ratio.

^b^Not applicable.

^c^CSSA: Comprehensive Social Security Assistance.

**Table 4:** Associations between perceived barriers to performing physical activity and low physical activity level.

Perceived barriers^a^	Round 1 (n=395)			Round 2 (n=370)	
	AOR^b^ (95% CI)	*P* value	AOR (95% CI)		*P* value
Do not have time	4.19 (1.54-11.40)	.01	0.99 (0.33-2.92)		.98
Lack of interest	1.06 (0.59-1.90)	.85	0.91 (0.50-1.66)		.75
Cannot find people to do physical activity together	1.07 (0.68-1.67)	.78	1.80 (1.03-3.16)		.04
Lack of physical capacity to do physical activity	3.34 (1.61-6.94)	.001	2.92 (1.48-5.76)		.002
Physical activity will cause pain and discomfort	2.04 (1.15-3.61)	.02	2.82 (1.55-5.16)		.001
Lack of space and facility to do physical activity at home	0.74 (0.48-1.14)	.17	2.03 (1.11-3.72)		.02
Concern about COVID-19 infection when doing physical activity	1.73 (1.10-2.72)	.02	0.75 (0.48-1.19)		.23
Closure of facilities due to COVID-19 and its control measures	0.97 (0.66-1.48)	.95	1.12 (0.69-1.80)		.65
Peers refused to do physical activity with you due to COVID-19	1.45 (0.96-2.19)	.08	0.78 (0.48-1.29)		.33

^a^Responses were categorized into disagree/neutral (reference category) and agree.

^b^AOR: adjusted odds ratio; adjusted for significant background characteristics listed in Table 3.

## Discussion

### Principal Findings

This was one of the first studies to track changes in the levels and barriers to performing PA among older adults before and after entering the “postpandemic era.” One of the strengths of this study is that it was based on a random and population-based sample. In response to the fifth wave of the COVID-19 outbreak, the Hong Kong government closed all sports centers and venues, and advised older adults to stay at home and limit their outdoor activities due to their vulnerability to COVID-19 [24]. Therefore, it was not surprising to observe that a sizeable proportion of older adults had a low PA level (over 40%), which was much higher than that reported at the time before COVID-19 (about 20%) [6,7]. Despite an increasing trend in daily confirmed cases, we observed a significant increase in PA level among older adults in the second round of the survey. However, the proportion of older adults having a low PA level (27.8%) was still higher than that reported at the time before COVID-19 (20%) [6,7]. Therefore, efforts are needed to facilitate older adults to resume and increase PA in the “postpandemic era.”

### Comparison With Prior Work and Implications for Health Promotion

As compared to those in the first round, participants in the second round reported a much higher completion rate of the primary COVID-19 vaccine series (94% vs 78.7%) and the booster dose (58.9% vs 31.6%). Completing the primary COVID-19 series and/or the booster dose was associated with a higher PA level in both rounds. An increase in COVID-19 vaccination coverage might contribute to the increasing PA level in the second round. Vaccinated older adults might feel they are protected against COVID-19 and hence have fewer concerns to resume outdoor activities (eg, PA). Promoting COVID-19 vaccination and the booster dose might be useful strategies to improve PA among older adults in the future.

Our findings suggested some different barriers to performing PA applied to older adults in Hong Kong before and after the fifth wave of the outbreak. First, a higher proportion of older adults agreed that they did not have time for PA in the first round compared to those in the second round. Those who lacked time for PA were more likely to report a low PA level in the first round, but not in the second round. In Hong Kong, grandparents are the main caregivers of children [28,29]. A qualitative study suggested that the increased caring responsibility due to school closure and lack of childcare during the pandemic was a barrier to performing PA among older adults in the United Kingdom [21]. During the fifth wave of the COVID-19 outbreak, the Hong Kong government closed all playgroups, kindergartens, and primary schools between January 14 and April 21, 2022. During the same period, the Hong Kong government directed civil servants to work from home. However, most private organizations did not follow the work-from-home policy. Since civil servants only account for less than 2% of the entire working population in Hong Kong, most parents had to work as usual and could not take care of their children during school closure. As a result, the childcare responsibility suddenly increased among many grandparents, which reduced their availability to perform PA. Second, there was a significant decrease in concerns about COVID-19 infection over time. Such concern was associated with a lower PA level during the pandemic, but not in the “postpandemic era.” A decrease in daily confirmed cases and an increase in COVID-19 vaccination coverage might have reduced their concerns about COVID-19 infection. Third, lack of space and facilities for home fitness was a significant barrier to performing PA in the “postpandemic era,” but not in the first round of the survey. One possible explanation was that lack of time to perform PA caused by a sudden increase in childcare responsibility during school closure was an overwhelming barrier to performing PA during the outbreak. Therefore, although more participants indicated lacking space and a facility for home fitness in the first round of the survey, they might not consider it as a significant barrier. Health promotion in the “postpandemic era” should encourage older adults to perform home-based PA without extensive requirement of space or facilities, such as stationary aerobic exercise or weight/strength training. Although the Hong Kong government used a series of exercise videos on television to promote PA at home for older adults living in public housing estates during the pandemic [30], the efficiency of such programs in promoting PA for older adults should be further explored. In addition, facilitating older adults to form peer support groups is a useful strategy to promote PA in the “postpandemic era.” Forming such peer support groups was less feasible during the pandemic when group gatherings were prohibited [24]. Since the government already lifted the restriction on group social gatherings, health promotion in the “postpandemic era” should consider using such a strategy [31].

Some similar barriers have hindered older adults to perform PA both before and after the fifth wave of the COVID-19 outbreak. In this study, concern about PA capacity and that PA would cause pain and discomfort were associated with a lower PA level. These concerns were also barriers to performing PA in this group before the time of COVID-19 [7,20]. Sport scientists should introduce feasible options of PA suitable for older adults. Testimonials of older adults on how they overcome these physical and environmental barriers and stay active might also be useful. Studies suggested that communication among Chinese older adults is very effective due to the high level of rapport among people of similar age [32]. Chinese older adults prefer to seek information and opinion from their peers, which are considered to be more credible than other information sources [32].

In contrast to our hypothesis, the closure of facilities due to COVID-19 and its control measures were not significantly associated with the PA level in the first or the second round. A previous study suggested that older adults in Hong Kong changed their mode of PA to cope with the COVID-19 control measures. For example, older adults reduced swimming or going to the gym for exercise, but increased stretching, brisk walking, or other activities that rely less on access to sports facilities [10]. Such adaptations might have mitigated the impact caused by the closure of sports facilities.

### Limitations

This study had some limitations. First, it was a major limitation that we did not assess the functional limitations of the participants. In Hong Kong, 16.3% of community-dwelling older adults had a functional limitation [33]. Older adults with functional limitation had lower PA levels. Second, we did not gather more information on family members of the participants due to the limited length of the questionnaire. Family members would have an influence on participants’ PA level (eg, whether family members can share the childcare responsibility during school closure). Third, PA was self-reported. We did not use accelerometers or wearable devices to assess PA due to feasibility and resource constraints. This raised concerns about reliability and recall bias. Fourth, due to the limited length of the questionnaire, some determinants of PA among older adults were not covered by the study (eg, perceived benefits and self-efficacy of PA). Fifth, we did not measure participants’ physical capacity to perform PA. Sixth, compared to census data, people who were 75 years or above were undersampled in this study [34]. However, the distributions of gender and age were similar to those of recent random telephone surveys among community-dwelling older adults [35]. Furthermore, nonresponses would cause selection bias. We were not able to obtain characteristics of those who refused to participate, which might be different from those of the participants. Our response rate was comparable to those of previous random telephone surveys targeting older adults in Hong Kong [32,35,36]. Last but not least, this was a cross-sectional study and could not establish causal relationships.

### Conclusion

The level of PA increased significantly after the drop of daily confirmed COVID-19 cases and the relaxation of COVID-19 control measures among older adults in Hong Kong. Different factors influenced older adults’ PA level during and after the fifth wave of the COVID-19 outbreak. Barriers to performing PA, such as perceived lack of space and a facility to perform PA at home, concerns about COVID-19 infection during PA, closure of facilities, and refusals made by peers to perform PA reduced significantly over time. Regular monitoring of the PA level and its associated factors should be conducted to guide health promotion and policy-making. Sport scientists should introduce suitable options for older adults with inadequate physical capacity or having functional limitations. In the “postpandemic era,” reactivating peer support groups and promoting home-based PA may increase the PA level among older adults. Health authorities should be aware of the negative impact of school closure on PA among older adults. If school closure has to be implemented in future waves of a COVID-19 outbreak, introducing PA options suitable for older adults to perform with their grandchildren may be helpful to alleviate its negative influence.

## Appendix

Multimedia Appendix 1 STROBE checklist for cross-sectional study.

Multimedia Appendix 2 The English and Chinese versions of the questionnaires used in the telephone survey.

Multimedia Appendix 3 Changes in physical activity among subgroups of participants.

## Abbreviations

AOR: adjusted odds ratio
IPAQ-SF: International Physical Activity Questionnaire-Short Form
MET: metabolic equivalent of tasks
MVPA: moderate-to-vigorous physical activity
PA: physical activity
WHO: World Health Organization

## Data Availability

The data sets generated and/or analyzed during the current study are available from the corresponding author on reasonable request.
