# Supplementary material for: The Changes in Levels and Barriers of Physical Activity Among Community-Dwelling Older Adults During and After the Fifth Wave of COVID-19 Outbreak in Hong Kong: Repeated Random Telephone Surveys
Source: JMIR Aging. 2023 Jan 23;6:e42223. doi: 10.2196/42223 (PMC9947816; doi:10.2196/42223)
Supplement: Multimedia Appendix 2 [file aging_v6i1e42223_app2.docx]

Multimedia appendix 2

Questionnaire in English and Chinese

**Q1**係過去七日，你有幾多日曾經參與過最少十分鐘嘅劇烈體育活動？劇烈體育活動係指一啲會令你嘅呼吸比平時深而急速，難以講野，出好多汗或好攰嘅活動。譬如搬重野、行山、跑步、游水、打波或踩單車踩到好快。

Think about all the vigorous activities that you did in the last 7 days. Vigorous physical activities refer to activities that take hard physical effort and make you breathe much harder than normal. Think only about those physical activities that you did for at least 10 minutes at a time. During the last 7 days, on how many days did you do vigorous physical activities like heavy lifting, digging, hiking, running, swimming, playing basketball/football, or fast bicycling?

□_1_無 --- 請回答Q2 □_2_ 有 --- 每星期_____日--- 請回答Q1a

No – please answer Q2 Yes --- ____days per week

**Q1a** 係你有做劇烈體育活動嘅呢幾日，你通常每日會用幾多時間參與呢啲劇烈體育活動呢？

How much time did you usually spend doing vigorous physical activities on one of those days?

_____小時_____分鐘

_____ hours _____ minutes per day

**Q2** 係過去七日，你有幾日曾經參與最少十分鐘嘅中度體力活動呢？中度體力活動係指一啲會令你呼吸比平時快一啲，出汗或有啲攰嘅活動，但唔會影響你講野。譬如太極、伸展操、晨運、做家務 (如: 掃地、拖地、買餸、洗車、一般園藝工作) 、搬輕野、慢慢踩單車、打乒乓波、跳舞、快步行，但唔包括步行

Think about all the moderate activities that you did in the last 7 days. Moderate activities refer to activities that take moderate physical effort and make you breathe somewhat harder than normal. Think about those physical activities that you did for at least 10 minutes at a time. During the last 7 days, on how many days did you do moderate physical activities like carrying light loads, bicycling at a regular pace, or doubles tennis? Do not include walking.

□_1_無 --- 請回答Q3 □_2_ 有 --- 每星期_____日--- 請回答Q2a

No – please answer Q3 Yes --- ____days per week

**Q2a** 係你有做中度體育活動嘅呢幾日，你通常每日會用幾多時間參與呢啲中度體力活動呢？

How much time did you usually spend doing moderate physical activities on one of those days?

_____小時_____分鐘

_____ hours _____ minutes per day

**Q3**係過去七日，你有幾日連續步行至少十分鐘？步行包括係工作嘅地方、屋企、出街同參與康樂體育活動時嘅步行或者散步。

Think about the time you spent walking in the last 7 days. This includes at work and at home, walking to travel from place to place, and any other walking that you have done solely for recreation, sport, exercise, or leisure. During the last 7 days, on how many days did you walk for at least 10 minutes at a time?

□_1_無 --- 請回答Q4 □_2_ 有 --- 每星期_____日--- 請回答Q3a

No – please answer Q4 Yes --- ____days per week

**Q3a** 係你有連續步行至少十分鐘嘅呢幾日，你通常每日會用幾多時間步行？

How much time did you usually spend walking on one of those days?

_____小時_____分鐘

_____ hours _____ minutes per day

**Q4**係過去七日，你每日有幾多時間係坐係到嘅？包括工作、屋企、睇電視、閱讀、搭車、及其他空閒嘅時間等。

This question is about the time you spent sitting on weekdays during the last 7 days. Include time spent at work, at home, while doing course work and during leisure time. This may include time spent sitting at a desk, visiting friends, reading, or sitting or lying down to watch television. During the last 7 days, how much time did you spend sitting on a week day?

每日_____小時_____分鐘

_____ hours _____ minutes per day

**Q5**請問你是否同意以下關於體力活動嘅講法

Do you agree with the following statements about physical activity

|  | 不同意  Disagree | 無意見  Neutral | 同意  Agree |
| --- | --- | --- | --- |
| a) 你無時間做運動  Do not have time to perform physical activity | 1 | 2 | 3 |
| b) 你無興趣做運動  Lack of interest to perform physical activity | 1 | 2 | 3 |
| c) 你揾唔到人同你一齊做運動  Cannot find people to do physical activity together | 1 | 2 | 3 |
| d) 你認為自己的體能不足唔無法做運動  Lack of physical capacity to do physical activity | 1 | 2 | 3 |
| e) 運動會導致身體疼痛或者其他嘅不適  Physical activity will cause pain and discomfort | 1 | 2 | 3 |
| f) 你擔心運動時感染新冠肺炎  Concern about COVID-19 infection when doing physical activity | 1 | 2 | 3 |
| g) 你做開運動嘅場所因為新冠肺炎疫情而關閉  Closure of facilities due to COVID-19 and its control measure | 1 | 2 | 3 |
| h) 經常同你一齊做運動嘅同伴因為新冠肺炎疫情而唔願意出嚟  Peers refused to do physical activity with you due to COVID-19 | 1 | 2 | 3 |
| i) 屋企裡面缺乏做運動嘅器材同地方  Lack of space and facility to do physical activity at home | 1 | 2 | 3 |

**基本情況**

Q6性別 (Sex assigned at birth)： □_1_男 (male) □_2_女 (female)

Q7年齡 (Age)： _______歲 (years)

Q8教育程度(Education level)

□_1_ 小學以下 (Below primary school)

□_2_ 小一至小六 (Primary school)

□_3_ 中一至中三 (Junior high school)

□_4_ 中四至中五或者預科 (senior high school)

□_5_專上或大學 (College or university)

□_6_ 大學以上 (Postgraduate)

□_7_ 其他，請註明______________ (Others, please be specific)

Q9你而家嘅婚姻狀況係？(What is your relationship status?)

□_1_ 未婚 (Single)

□_2_ 已婚 (Married)

□_3_ 分居/離婚 (Separated/divorced)

□_4_ 喪偶 (Widowed)

□_5_同居 (Cohabited with a partner)

□_6_ 其他，請註明_______________________ (Others, please be specific)

Q10你而家嘅工作情況係？(What is your current employment status?)

□_1_ 全職 (Full-time)

□_2_ 兼職 (Part-time)

□_3_ 退休 (Retired)

□_4_ 待業/失業 (Unemployed)

□_5_家庭主婦 (Housewife)

□_6_ 其他，請註明_______________________ (Others, please be specific)

Q11你而家是否獨居？(Are you living alone?)

□_1_是 (Yes)

□_2_否 (No)

Q12你每月平均家庭收入(即係計哂所有同你一齊住嘅屋企人嘅總收入)係邊個範圍？(What is your monthly personal income?)

□_1_ 20,000以下 (Below 20000)

□_2_ 20,000-39,999

□_3_ 40,000-59,999

□_4_ 60,000-79,999

□_5_ 80,000或以上 (80000 or above)

□_6_ 冇固定收入 (No fixed income)

□_7_ 拒絕透露 (Refuse to disclose)

Q13請問你有冇領取綜援？(Are you receiving comprehensive social security assistance?)

□_1_有 (Yes)

□_2_冇 (No)

Q14我地想了解一下你目前嘅健康狀況 (Do you have the following chronic conditions?)

|  | 有  Yes | 冇  No | 唔清楚  Do not know |
| --- | --- | --- | --- |
| 1高血壓  Hypertension | 1 | 2 | 3 |
| 2其他長期心血管疾病（例如心臟病、腦血管病等）  Chronic cardiovascular diseases | 1 | 2 | 3 |
| 3 慢性肺病（例如慢性阻塞性肺病、肺癌等）  Chronic lung diseases | 1 | 2 | 3 |
| 4慢性肝病（例如慢性肝炎、肝硬化、肝癌等）  Chronic liver diseases | 1 | 2 | 3 |
| 5 慢性腎病（例如慢性腎炎、慢性腎功能不全/腎衰竭、腎癌等）  Chronic kidney diseases | 1 | 2 | 3 |
| 6糖尿病  Diabetes Mellitus | 1 | 2 | 3 |

Q15你有無被確診患上新冠肺炎？(Did you have history of COVID-19?)

□_1_有 (Yes) □_2_ 無 (No) □_3_ 唔清楚 (Do not know)

Q16請問你有無打過新冠肺炎疫苗？(Did you receive COVID-19 vaccination?)

□_1_目前無 (No)

□_2_打過1針 (1 dose)

□_3_打過2針 (2 doses)

□_4_打過3針 (3 doses)

□_5_打過4針 (4 doses)

**問卷結束，感謝你嘅參與**

End of the questionnaire. Thank you for your support.
