# Supplementary material for: The Changes in Levels and Barriers of Physical Activity Among Community-Dwelling Older Adults During and After the Fifth Wave of COVID-19 Outbreak in Hong Kong: Repeated Random Telephone Surveys
Source: JMIR Aging. 2023 Jan 23;6:e42223. doi: 10.2196/42223 (PMC9947816; doi:10.2196/42223)
Supplement: Multimedia Appendix 3 [file aging_v6i1e42223_app3.docx]

Multimedia appendix 3. Changes in physical activity among subgroups of participants.

Table 1 Changes in PA level among different subgroups of participants

|  | Perceived a lack of physical capacity to performing PA | | | Did not perceive a lack of physical capacity to performing PA | | |
| --- | --- | --- | --- | --- | --- | --- |
|  | First  Round (n=40) | Second round  (n=42) | P values | First  Round (n=357) | Second round  (n=328) | P values |
| PA levels, n (%) |  |  |  |  |  |  |
| Low | 29 (72.5) | 22 (52.4) |  | 151 (42.5) | 84 (25.6) |  |
| Moderate | 9 (22.5) | 12 (28.6) |  | 144 (40.6) | 149 (45.4) |  |
| High | 2 (5.0) | 8 (19.0) | .08 | 60 (16.9) | 95 (29.0) | .001 |
| Met-minutes/week, median (IQR) | 198  (0, 840) | 693  (292.75, 1925.25) | .005 | 939  (420, 2079) | 1735.5  (840, 3461.25) | <.001 |
| Minutes of MVPA per week, median (IQR) | 0  (0, 110) | 45  (0, 270) | .08 | 140  (0, 420) | 260  (90, 603.75_ | <.001 |
|  | Perceived PA would cause pain and discomfort | | | Did not perceive PA would cause pain and discomfort | | |
|  | First  Round (n=60) | Second round  (n=56) | P values | First  Round (n=335) | Second round  (n=314) | P values |
| PA levels, n (%) |  |  |  |  |  |  |
| Low | 37 (61.7) | 27 (48.2) |  | 143 (42.7) | 79 (25.2) |  |
| Moderate | 17 (28.3) | 17 (30.4) |  | 136 (40.6) | 144 (45.9) |  |
| High | 6 (10.0) | 12 (21.4) | .18 | 56 (16.7) | 91 (29.0) | <.001 |
| Met-minutes/week, median (IQR) | 556  (0, 1014.75) | 873.25  (364.5, 2410.5) | .02 | 939  (398, 2076) | 1738.25  (840, 3425.25) | <.001 |
| Minutes of MVPA per week, median (IQR) | 51  (0, 210) | 130  (0, 420) | .08 | 130  (0, 420) | 265  (90, 600) | <.001 |

Table 2 Comparison of PA levels between different subgroup of participants at the same time point

|  | First round | | | Second round | | |
| --- | --- | --- | --- | --- | --- | --- |
|  | Perceived a lack of physical capacity    (n=40) | Did not perceive a lack of physical capacity  (n=357) | P values | Perceived a lack of physical capacity    (n=42) | Did not perceive a lack of physical capacity  (n=328) | P values |
| PA levels, n (%) |  |  |  |  |  |  |
| Low | 29 (72.5) | 151 (42.5) |  | 22 (52.4) | 84 (25.6) |  |
| Moderate | 9 (22.5) | 144 (40.6) |  | 12 (28.6) | 149 (45.4) |  |
| High | 2 (5.0) | 60 (16.9) | <.001 | 8 (19.0) | 95 (29.0) | <.001 |
| Met-minutes/week, median (IQR) | 198  (0, 840) | 939  (420, 2079) | <.001 | 693  (292.75, 1925.25) | 1735.5  (840, 3461.25) | <.001 |
| Minutes of MVPA per week, median (IQR) | 0  (0, 110) | 140  (0, 420) | <.001 | 45  (0, 270) | 260  (90, 603.75_ | <.001 |
|  | First round | | | Second round | | |
|  | Perceived PA would cause pain and discomfort  (n=60) | Did not perceive PA would cause pain and discomfort  (n=335) | P values | Perceived PA would cause pain and discomfort    (n=56) | Did not perceive PA would cause pain and discomfort  (n=314) | P values |
| PA levels, n (%) |  |  |  |  |  |  |
| Low | 37 (61.7) | 143 (42.7) |  | 27 (48.2) | 79 (25.2) |  |
| Moderate | 17 (28.3) | 136 (40.6) |  | 17 (30.4) | 144 (45.9) |  |
| High | 6 (10.0) | 56 (16.7) | <.001 | 12 (21.4) | 91 (29.0) | <.001 |
| Met-minutes/week, median (IQR) | 556  (0, 1014.75) | 939  (398, 2076) | <.001 | 873.25  (364.5, 2410.5) | 1738.25  (840, 3425.25) | <.001 |
| Minutes of MVPA per week, median (IQR) | 51  (0, 210) | 130  (0, 420) | <.001 | 130  (0, 420) | 265  (90, 600) | <.001 |
